# Insights Into the Binding Mechanism of GC7 to Deoxyhypusine Synthase in *Sulfolobus solfataricus*: A Thermophilic Model for the Design of New Hypusination Inhibitors

**DOI:** 10.3389/fchem.2020.609942

**Published:** 2020-12-17

**Authors:** Mattia D'Agostino, Stefano Motta, Alice Romagnoli, Patrick Orlando, Luca Tiano, Anna La Teana, Daniele Di Marino

**Affiliations:** ^1^Department of Life and Environmental Sciences, Polytechnic University of Marche, Ancona, Italy; ^2^Department of Earth and Environmental Sciences, University of Milano-Bicocca, Milan, Italy; ^3^New York-Marche Structural Biology Center (Ny-Masbic), Polytechnic University of Marche, Ancona, Italy

**Keywords:** DHS, hypusination, GC7, translation, metadynamics simulation, eIF5A, initiation factor 5A

## Abstract

Translation factor 5A (eIF5A) is one of the most conserved proteins involved in protein synthesis. It plays a key role during the elongation of polypeptide chains, and its activity is critically dependent on hypusination, a post-translational modification of a specific lysine residue through two consecutive enzymatic steps carried out by deoxyhypusine synthase (DHS), with spermidine as substrate, and deoxyhypusine hydroxylase (DOHH). It is well-established that eIF5A is overexpressed in several cancer types, and it is involved in various diseases such as HIV-1 infection, malaria, and diabetes; therefore, the development of inhibitors targeting both steps of the hypusination process is considered a promising and challenging therapeutic strategy. One of the most efficient inhibitors of the hypusination process is the spermidine analog N1-guanyl-1,7-diaminoheptane (GC7). GC7 interacts in a specific binding pocket of the DHS completely blocking its activity; however, its therapeutic use is limited by poor selectivity and restricted bioavailability. Here we have performed a comparative study between human DHS (hDHS) and archaeal DHS from crenarchaeon *Sulfolobus solfataricus* (aDHS) to understand the structural and dynamical features of the GC7 inhibition. The advanced metadynamics (MetaD) classical molecular dynamics simulations show that the GC7 interaction is less stable in the thermophilic enzyme compared to hDHS that could underlie a lower inhibitory capacity of the hypusination process in *Sulfolobus solfataricus*. To confirm this hypothesis, we have tested GC7 activity on *S. solfataricus* by measuring cellular growth, and results have shown the lack of inhibition of aIF5A hypusination in contrast to the established effect on eukaryotic cellular growth. These results provide, for the first time, detailed molecular insights into the binding mechanism of GC7 to aDHS generating the basis for the design of new and more specific DHS inhibitors.

## Introduction

Protein synthesis represents the final step of gene expression and, being one of the most energy-consuming process in cells, not surprisingly, it is highly regulated in all domains of life.

Regulation can be exerted either on the whole translation process or on specific mRNAs, and it can be mediated by different cis-acting elements present on the mRNAs, as well as by trans-acting factors (Sonenberg and Hinnebusch, 2009; Bhat et al., 2015).

Several of the regulatory proteins belong to the group of translation factors (Bhat et al., 2015), and one of the most important ones is the protein called Initiation Factor 5A in Eukarya and Archaea (eIF5A/aIF5A) and EF-P in Bacteria (Dever et al., 2014; Rossi et al., 2014; Benelli et al., 2017).

IF5A belongs to the small group of the universally conserved translation factors (Kyrpides and Woese, 1998). It is an abundant, acidic protein, which plays a fundamental role by promoting recovery of translation on ribosomes, which are stalling during synthesis of proteins containing particular sequences (for example, stretches of polyproline) (Park and Wolff, 2018). In addition to its function in translation, IF5A has been proposed to play other roles (Park et al., 2010; Bassani et al., 2019).

In order to correctly perform its function in translation, IF5A must undergo a unique and characteristic posttranslational modification called hypusination in Eukarya and Archaea, and β-lysinylation in Bacteria (Rajkovic and Ibba, 2017; Park and Wolff, 2018).

For the aims of this work, we will focus only on the eukaryal and the archaeal proteins.

**Figure 1:**
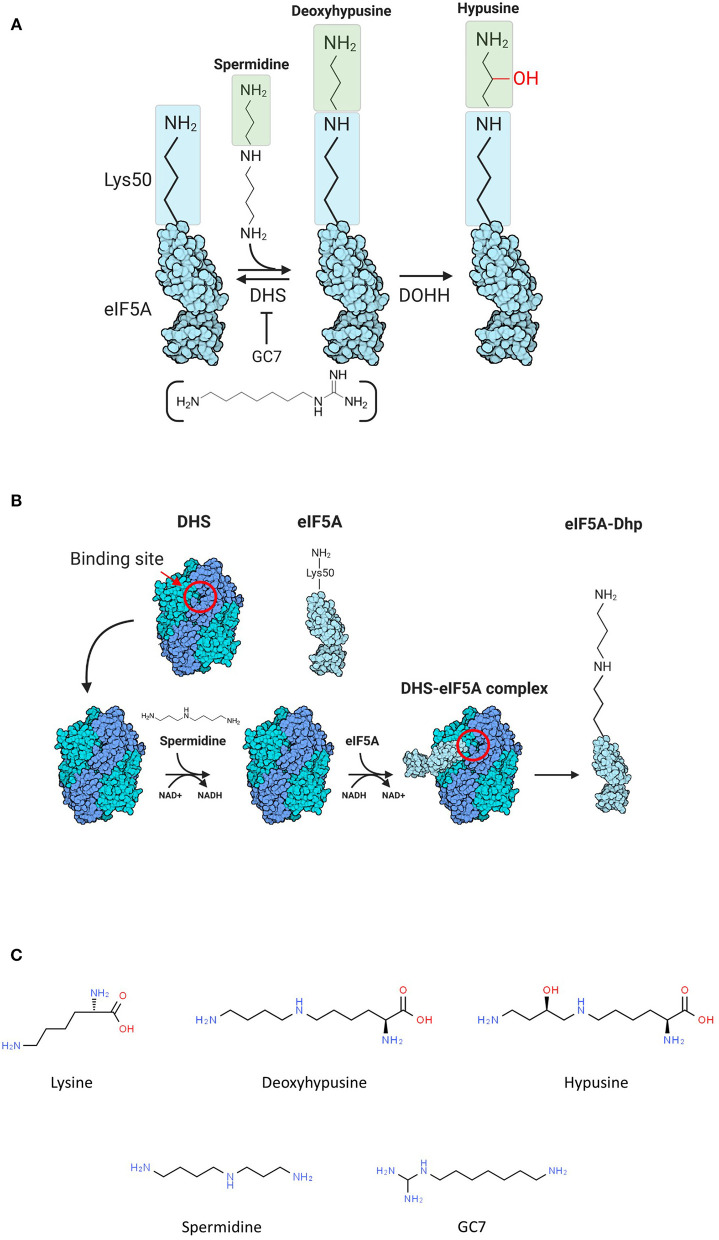
**(A)** Scheme of hypusination reaction. **(B)** Scheme of the first part of hypusination reaction. The binding site of deoxyhypusine synthase (DHS) is reported (red circle). **(C)** Chemical formulations of molecules involved in the hypusination pathway.

Hypusination consists in the transformation of a conserved lysine residue (Lys 50 in human eIF5A) into a nonstandard amino acid called hypusine using spermidine as a substrate (Park et al., 1981; Park and Wolff, 2018), and this modification pathway, as characterized in Eukarya, is carried out by two enzymes: deoxyhypusine synthase (DHS) and deoxyhypusine hydroxylase (DOHH) (Figure 1A). The first step of the reaction is characterized by the transfer of the aminobutyl moiety, produced by the cleavage of spermidine, on the conserved lysine with the formation of deoxy-hypusine (Figure 1B). The DOHH catalyzes the oxidation of the deoxy-hypusine converting it to hypusine (Figure 1A) (Park et al., 1981).

The modification pathway is conserved in all Eukarya, while Archaea show some heterogeneity (Bartig, 1992; Prunetti et al., 2016). In some organisms, aIF5A is hypusinated; in others, only the deoxyhypusinated version is present and very few contain both versions of the protein. A DHS gene has been found in all archaeal genomes sequenced so far, while no DOHH homolog has been identified. Nevertheless, several archaeal strains contain the hypusine modification, leaving the question of how this posttranslational modification occurs in these organisms unanswered (Park and Wolff, 2018).

Our group has demonstrated that aIF5A from the hyperthermophilic crenarchaeon *Sulfolobus solfataricus* is hypusinated, as its eukaryotic counterpart and that *S. solfataricus* DHS (aDHS), that shares with the human homolog 31 and 52% of amino acid identity and similarity, respectively, interacts with aIF5A catalyzing the formation of deoxyhypusine, suggesting that the first step of hypusination is conserved in this organism (Bassani et al., 2018) (Figure 1B). Recently, crystal structures of two eukaryotic, *H. sapiens* (Umland et al., 2004; Wator et al., 2020) and *T. brucei*, and one archaeal, *P. horikoshii* (Chen et al., 2020), DHS enzymes have been solved. All three enzymes share a similar folding being organized in tetramers composed by four monomers of ~40 kDa, with two active sites located at the dimer interface (Umland et al., 2004; Chen et al., 2020). In addition, the structure of the human enzyme in complex with different ligands like spermidine, spermine, putrescine, NAD+, and GC7 is also available (Umland et al., 2004; Wator et al., 2020). Spermidine binds DHS into a small cavity composed of amino acids with specific physicochemical properties able to establish a characteristic network of interaction with the ligand. Indeed, the two terminal groups of spermidine interact with three acidic residues of DHS (Asp243-, Asp316-, and Glu323) anchoring it into the narrow binding pocket (Lee et al., 2001; Wator et al., 2020).

**Figure 2:**
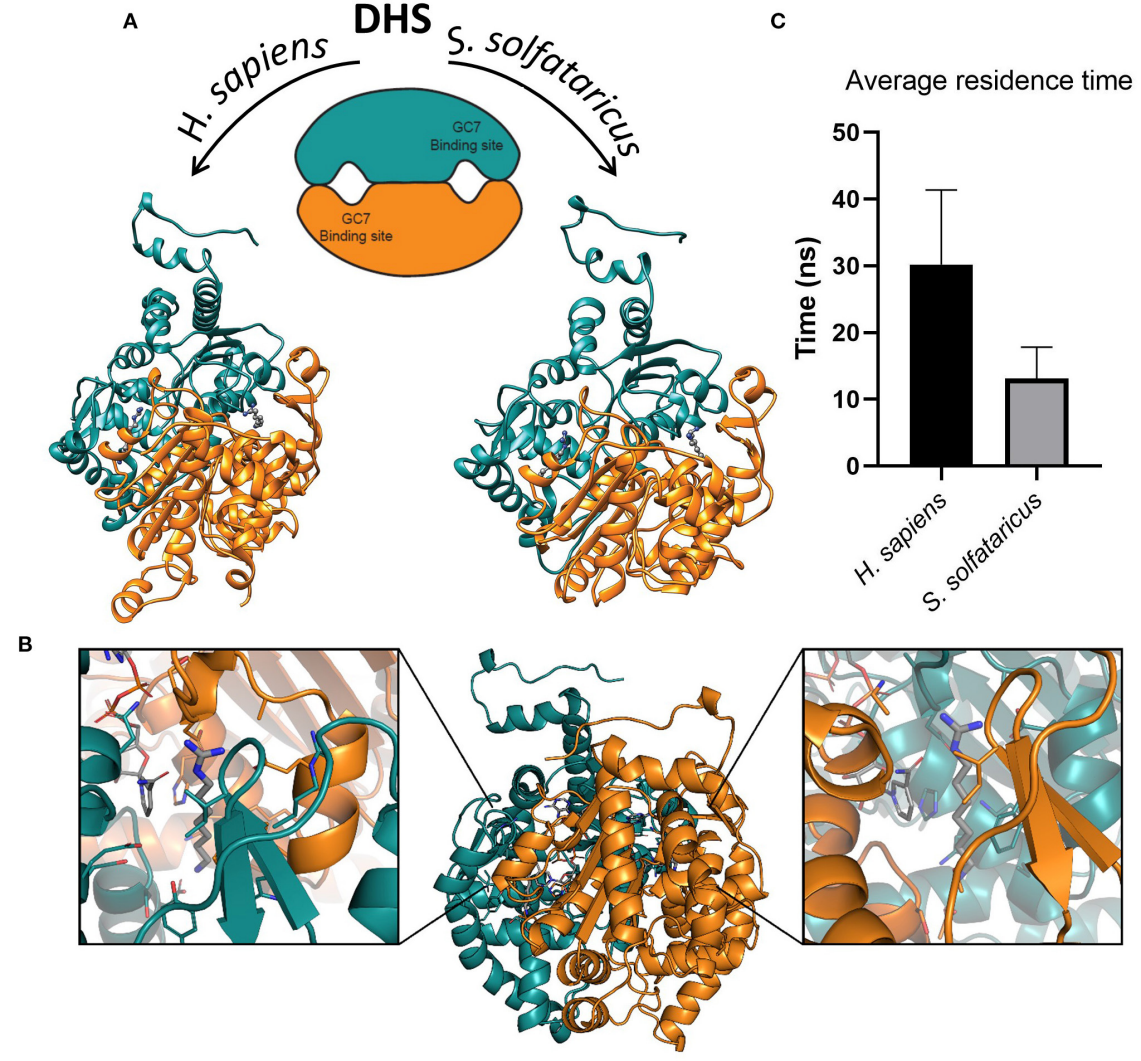
**(A)** Structures of deoxyhypusine synthase in *Homo sapiens* and *Sulfolobus solfataricus*. Schematic and three-dimensional representations of symmetric dimeric structure of *H. sapiens* (PDBID:1RQD) and *S. solfataricus* DHS obtained through homology modeling. The two GC7 binding sites are shown in the interfaces between the two monomers. The two molecules of GC7 are present inside the binding pockets in both models. **(B)** Symmetric binding sites in the *H. sapiens* DHS dimer. Binding of GC7 occurs at the interface between the two subunits (orange and eastern-blue in figure). GC7 is shown in gray thick sticks, sidechain of residues within 5 Å from GC7 in sticks colored according to the subunit, and NAD molecule in gray sticks. **(C)** Average residence time measured for the unbinding of GC7 from human and archaeal DHS in metadynamic (MetaD) simulations. Average residence times are significantly different (*p* < 0.05).

The eIF5A structure from several organisms has also been solved (Dever et al., 2014), and despite the bunch of structural and biochemical information available, the three-dimensional (3D) organization of the DHS–eIF5A complex is still unknown. The strong geometric complementarity of DHS and eIF5A structures provides some useful clues for potential organization of the complex. In fact, the DHS binding pocket for spermidine is a narrow cavity, whereas the conserved lysine on eIF5A is located at the peak of a loop connecting two β-strands (Figure 2B).

Human eIF5A exists in two isoforms, eIF5A1 and eIF5A2, and both have been related to several diseases. In particular, both isoforms of eIF5A are overexpressed in many types of cancer [for review, see Mathews and Hershey (2015)] and in various others diseases such as HIV-1 infection (Bevec et al., 1996), malaria (Kaiser et al., 2007), and diabetes (Maier et al., 2010).

The involvement in pathogenesis together with the high specificity and functional relevance of the hypusination reaction have prompted researchers to consider eIF5A and its modification pathway as an important and promising therapeutic target stimulating the design and development of eIF5A inhibitors able to target the hypusination process (Olsen and Connor, 2017; Turpaev, 2018), including the DHS–eIF5A complex formation (Figure 1B).

Different molecules have already been developed as specific inhibitors of both DHS (Jakus et al., 1993; Nakanishi and Cleveland, 2016; Schultz et al., 2018) and DOHH (Hoque et al., 2009; Olsen and Connor, 2017), but those targeting DHS are characterized the best. To date, no inhibitors targeting the DHS–eIF5A complex formation have been discovered.

The most powerful DHS inhibitor, among the various spermidine analogs is N1-guanyl-1,7-diaminoheptane (GC7) (Figure 1C), a compound showing a Ki = 10 nM, which is ~500 times lower than the Km of the physiological spermidine (Jakus et al., 1993).

The use of GC7, alone or in combination with other drugs, was demonstrated to inhibit the growth of various mammalian cells (Nakanishi and Cleveland, 2016; Schultz et al., 2018; Martella et al., 2020). However, GC7 and other DHS inhibitors are not sufficiently selective giving rise to several side effects. Furthermore, the bioavailability of these compounds is restricted by physiological polyamine oxidases present in the blood. For these reasons they are not used in clinical trials (Turpaev, 2018) making possible further studies concerning the inhibition of the DHS function.

In light of the strong similarity between eukaryal and archaeal proteins, we thought that the archaeal could represent a model system that is able to provide structural and biochemical information, which can turn useful in the design of new inhibitors of both hypusination process and DHS–eIF5A complex formation. Moreover, we believe that understanding of the molecular details of the DHS inhibition in extremophiles such as *S*. *solfataricus* provides further insights for a more precise drug design of molecules that can be used to treat different pathologies.

In order to address this issue, we investigated the interaction between DHS and its inhibitor GC7 by comparing the human and *Sulfolobus* system. We started from the observation that, despite the enzyme conservation, GC7 shows a different behavior on different archaeal organisms (Jansson et al., 2000).

Here we have used a combination of advanced computational approaches and experimental techniques to analyze the different features of the binding mode of GC7 into the active site in both *H. sapiens* and *S. solfataricus* DHS (i.e., hDHS and aDHS).

Results from metadynamic (MetaD) simulations highlighted a different stability of the two GC7–DHS complexes, which is due to specific interaction networks established within the binding sites. Interaction of GC7 with aDHS is significantly less stable compared to hDHS, and this result was validated by *in vivo* experiments on *S. solfataricus* cells whose growth was unaffected by the presence of GC7.

This comparative and multidisciplinary study provided an in-depth characterization of the molecular mechanism of interaction of GC7 with both human and archaeal DHS paving the way for the design of new, specific, and more sensitive DHS inhibitors.

## Materials and Methods

### *Sulfolobus solfataricus* Cell Growth

*S. solfataricus* P2 cultures were grown in liquid Brock's medium (Brock's salts supplemented with 0.2% N-Z-amine, 0.2% sucrose, pH 3.0) in a shaking water bath at a speed of 150 rpm at the temperature of 348 K (75°C). Growth was monitored by measuring the optical density at 600 nm (OD600). For a typical experiment, an *S. solfataricus* P2 culture with a starting OD600 of 0.05 was split into six different flasks of 10 ml each, and GC7 (Sigma-Aldrich) was added in the following concentrations: 0 (control), 10, 50, 100, 250, and 500 μM respectively. OD600 measurements were taken at 0 hour (h), 15, 24, 48, 72, and 96 h. At a time point of 48 h, a 1-ml aliquot from each sample was harvested and centrifuged. The resulting cell pellets were washed three times with fresh Brock's medium and then resuspended with a resuspension buffer [20 mM Tris/HCl pH 7.8, 10 mM Mg(CH_3_COO)_2_, 40 mM NH_4_Cl, 6 mM β-mercaptoethanol]. Cells were lysed by six freeze and thaw cycles, and total protein concentration of each sample was determined using the Bradford reagent (Sigma-Aldrich). The levels of hypusinated aIF5A in each lysate sample were analyzed by Western blot.

### Western Blot

Western blot analysis was performed as previously described (Bassani et al., 2018) with some modifications. Briefly, 15 μg of total proteins for each sample was separated on SDS-15% polyacrylamide gel using standard protocols and then transferred onto a 0.2-μm nitrocellulose membrane (GE Healthcare) using wet transfer blotting apparatus. Protein transfer was performed at 100 V for 30 min in a transfer buffer (25 mM Tris, 192 mM glycine, 20% methanol). Nonspecific binding was blocked using 5% nonfat milk. The membranes were probed overnight at 4°C, either with anti-aIF5A (used at a 1:5,000 dilution in TBS-Tween containing 5% of nonfat milk) or with anti-hypusine antibody (Millipore). The detection of primary antibodies was obtained by horseradish peroxidase (HRP)-conjugated anti-rabbit IgG (Cell Signaling Technology), using the enhanced chemiluminescent reagent (EuroClone). The images were visualized with a BioRad ChemiDoc™ MP Imaging System. The quantification of the signals was obtained by the ImageLab™ software (Biorad).

### Analysis of GC7 Uptake

The evaluation of the presence of GC7 inside the cells was obtained through the dansyl-chloride method as previously described (Ahmed et al., 2017), with some modifications. Briefly, 1 ml of cell cultures grown in the absence and in the presence of 500 μM of GC7 were collected by centrifugation after 72 h; the resulting pellets were washed three times with fresh Brock's medium to avoid false positives due to free GC7 in the medium. The pellets (~40 mg) were extracted in 5% cold perchloric acid. Sample extracts were centrifuged at 27,000 g for 10 min. Dansyl derivatization was obtained, 200 μl of extracted sample was mixed with 200 μl of saturated sodium carbonate (100 mg/ml) and 400 μl of dansyl chloride in acetone (10 mg/ml). After vortexing, the mixture was incubated overnight at room temperature in the dark. Excess dansyl chloride was removed by adding 100 μl of L-proline (100 mg/ml) followed by incubation for 30 min at room temperature in the dark. The dansylated polyamines were further extracted with 350 μl of toluene. The organic phase containing polyamines and GC7 was evaporated using a speed-vac, and the residue was dissolved in 500 μl of methanol. The dansylated polyamines were separated by HPLC (YL Instrument 9300, Amaze instrument, Uttar Pradesh, India) equipped with a fluorescence detector (Nanospace-SI2, Shiseido) and a column Kinetex C18 100 A, 250 × 4.6 mm, 5 mm (Phenomenex, Torrance, CA, USA). For identification of GC7 and endogenous spermidine, standards were used (10 μl of 100 μM GC7 and 10 μl of 20 μM spermidine).

### Molecular Dynamics Simulations

Crystal structures for *H. sapiens* DHS in complex with GC7 inhibitor were obtained from the Protein Data Bank (PDB) in its homodimeric state (Umland et al., 2004) (1RQD). A homology model of the *S. solfataricus* DHS was built using an HHpred toolkit (https://toolkit.tuebingen.mpg.de/tools/hhpred) (Söding et al., 2005) selecting as template the X-ray structure of the *H. sapiens* DHS (PDBID:1RQD). The overall quality of the model was assessed with PROCHECK (Laskowski et al., 1993), which provides information about the stereo-chemical quality, and ProSA validation method (Wiederstein and Sippl, 2007), which evaluates model accuracy and statistical significance with a knowledge-based potential. The main results are reported in Supplementary Figure 1.

Both structures were then pre-processed for simulation with the Schrodinger's Protein Preparation Wizard tool (Madhavi Sastry et al., 2013): hydrogen atoms were added, all water molecules were removed, C and N terminal capping was added, disulfide bonds were assigned, and residue protonation states were determined by PROPKA (Bas et al., 2008) at pH = 7.0. Each system was then solvated in a cubic box with TIP3P water molecules and neutralized with Na^+^/Cl^−^ ions using the GROMACS (Abraham et al., 2015) preparation tools. The minimal distance between the protein and the box boundaries was set to 14 Å. Simulations were run using GROMACS 2018 with Amber ff14SB force-field (Maier et al., 2015). The parameter for NAD molecule was retrieved from amber library (Walker et al., 2002), while GC7 inhibitor was parameterized using GAFF (Wang et al., 2006). Charges were calculated with the restricted electrostatic potential (RESP) method (Bayly et al., 1993) at HF/6-31G^*^ after *ab initio* optimization. A multistage equilibration protocol, similar to the one applied in Motta et al. (2018) was applied to all simulations to provide a reliable starting point for the production. The system was subjected to a 2,000-step of steepest descent energy minimization with positional restraints (2,000 kJ mol^−1^ nM^−2^) on backbone and ligand atoms. Subsequently, MD simulation in an NVT ensemble was used to heat the system from 0 to 100 K in 1 ns with restraints lowered to 500 kJ mol^−1^ nM^−2^. Temperature was controlled by the Berendsen thermostat (Berendsen et al., 1984) with a coupling constant of 0.2 ps. The system was then heated up to 300 K (27°C) [348 K (75°C) in *S. solfataricus*] in 2 ns during an NPT simulation with restraint lowered to 200 kJ mol^−1^ nM^−2^ using the V-rescale thermostat (Bussi et al., 2007) with a coupling constant of 0.1 ps. Pressure was set to 1 bar with the Parrinello–Rahman barostat (Parrinello and Rahman, 1981) with a coupling constant of 2 ps. A time step of 1.0 fs was used during these steps, together with the LINCS algorithm (Hess et al., 1997) to constrain all the bonds.

Finally, the system was equilibrated in two stages of NPT simulations of 5 ns each, with backbone restraints lowered to 100 and 50 kJ mol^−1^ nM^−2^ respectively. The timestep was increased to 2.0 fs in these stages and during all the production runs. The particle mesh Ewald (Darden et al., 1993) method was used to treat the long-range electrostatic interactions with the cutoff distances set at 12 Å. Productions runs were conducted in NPT for 600 ns for both the *H. sapiens* and *S. solfataricus* systems.

### Metadynamics Simulations

Metadynamics is an enhanced sampling method based on the introduction of a history-dependent bias on a small number of suitably chosen collective variables (Laio and Parrinello, 2002; Laio and Gervasio, 2008; Bussi and Branduardi, 2015). A large number of studies was carried out to elucidate the binding/unbinding process of ligand/protein systems (Limongelli et al., 2010; Casasnovas et al., 2017; Provasi, 2019). More recently, infrequent metadynamics was introduced to study the unbinding kinetic (Tiwary and Parrinello, 2015; Tiwary et al., 2015; Wang et al., 2018). In a similar manner, here, we used metadynamics to speed-up the unbinding process, using the number of contacts between the binding site residues of DHS (residues at 4 Å from the native pose) and GC7 as unique CV, and performing 30 replicas to obtain a statistics on the “average residence time” of the ligand in metadynamics simulation. Hills with a height of 0.2 kJ mol^−1^ and a width of 2.0 were deposited every 2.0 ps at 300 K (27°C) for both systems. The number of contacts in the native pose was around 650 for both the *H. Sapiens* and *S. solfataricus* systems, and we considered the ligand as exited from the binding site when the number of contacts reaches the number of 200. This number is small enough to guarantee the overcoming of the energetic barrier splitting the bound and the unbound states. The residence time in MetaD calculation was computed as the average time required to observe the unbinding (number of contacts <200) in the different replicas. A change in this cutoff value does not appear to affect the result of the analysis (data not shown).

## Results

### Comparative Analysis of the hDHS and aDHS Structure

GC7 is the most potent inhibitor of DHS that represents the first enzyme involved in the two steps of the hypusination reaction. Although this molecule is effective in blocking the growth of various eukaryotic cell lines (Kaiser, 2012; Caraglia et al., 2013; Mathews and Hershey, 2015), on the other hand, it seems to have a slightly different effect in Archaea (Jansson et al., 2000), even if the enzyme is extremely conserved (Bassani et al., 2018). The structural and dynamical reasons of this discrepancy in the GC7 activity are still unknown. In order to investigate at the molecular level the interaction between GC7 and the two conserved enzyme (i.e., hDHS and aDHS), we have first built the three-dimensional (3D) structure of the DHS from *S. solfataricus* (Figure 2A, right side) using as template the 3D structure from *H. sapiens* DHS (Umland et al., 2004) (Figure 2A, left side).

As reported in different DHS X-ray structures (Umland et al., 2004; Tanaka et al., 2020; Wator et al., 2020), the binding of GC7 occurs at the interface between the two subunits of the tetramer, in a symmetric mode that leads to the formation of two binding sites for each dimer (Figure 2B). Interestingly, GC7 interacts with both monomers and with the NAD molecule that is located in a conserved pocket close to the GC7 binding site. Based on the hDHS X-ray structures bound to GC7 (Umland et al., 2004), we developed a structural model for aDHS. The GC7 binding orientation in aDHS was obtained starting from the position in hDHS and minimizing the structure. A careful equilibration protocol was introduced before the MD simulation to avoid artifacts due to a bad ligand starting position. As a result of this procedure, the ligand orientation remained unaltered, and only a few residue sidechains changed their conformation.

An in-depth comparative structural analysis of the GC7 binding site in the X-ray of hDHS and in the aDHS model highlights few differences in the residues composing the narrow pocket (Supplementary Figure 2). At the level of the guanidino group of GC7 into an active site, only two residues are different between hDHS and aDHS (Supplementary Figures 2A,B). A critical mutation is the hDHS Asp316 residue substituted by Leu272 in aDHS. The strongest differences in the binding site are located in correspondence of the GC7 amine group (Supplementary Figures 2C,D). Here the most critical substitution is Asp243 present in hDHS (forming a salt bridge with GC7) that is substituted by Thr200 in aDHS that does not allow the formation of a specific electrostatic interaction. Overall, only 10 over 25 residues change between hDHS and aDHS; most of them do not significantly alter the global shape and the general chemical properties of the pocket.

### Evaluation of GC7 Binding Stability in hDHS and aDHS Through Metadynamic Simulations

In order to characterize the binding stability of GC7 in hDHS and aDHS, we designed a MetaD protocol to evaluate the unbinding mechanism of GC7 from the conserved DHS binding pocket. We chose the number of contacts between the residues belonging to the binding site of both hDHS and aDHS and GC7 as CV to describe the energetic and structural–dynamical features of the GC7 binding stability. We have performed 30 replicas of MetaD simulation to collect a significant number of unbinding events allowing us to extract the molecular descriptors of GC7-hDHS/aDHS interaction. The simulations were stopped when the number of contacts between ligand and binding site residues were <200. This cutoff value corresponds to a confident prediction of the unbound state of the ligand as shown in two examples in Supplementary Figure 3. In this conformation, the GC7 center of mass (COM) drifted from the native bound state of about 11 Å.

An important parameter to evaluate the stability of a protein-ligand complex can be identified in the residence time of the ligand into the protein binding site. The trajectories of the GC7 unbinding obtained through MetaD simulations allowed us to observe the entire exit path of the ligand and also to compare the different residence time of the ligand into the hDHS and aDHS binding sites. Higher residence times is expected for the more stable complex based on the number and type of interactions established by the ligand (i.e., GC7) with the residues composing the protein binding pocket. Results of these calculations are shown in Figure 2C. Surprisingly, GC7 showed a significantly higher residence time in the *H. sapiens* DHS compared to the *S. solfataricus* one. The analysis of the unbinding mechanisms revealed two different unbinding pathways for hDHS (Supplementary Figure 3). Path A was sampled in most of the replicas (70%). Interestingly, the replicas following path A present a similar behavior, evolving through the same intermediate state with the amine group of GC7 involved in a salt bridge with Glu136 and Glu137. On the other side, in the replicas evolving through path B, GC7 leaves the pocket from the other side, and the amine group starts to interact with Glu180 (Supplementary Figure 4A). Notably, the guanidino group of GC7 forms a salt bridge with the same two residues involved in path A (i.e., Glu136 and Glu137). Similar pathways were also found during the aDHS MetaD simulations, with path A found in most of the replicas (70%). Due to the substitution in aDHS of Glu136 and Glu137 with Asp95 and His96, when GC7 leaves the binding pocket following the path A, no stable conformation was identified, and the exit occurs in a single passage in most of the replicas. On the contrary, replicas following path B always evolve forming an interaction with Glu141 (Glu180 in hDHS), but the interaction lasts for less time, and the GC7 is rapidly released in the solvent (Supplementary Figure 4B).

Although the two DHS enzymes share a significant sequence identity and similarity (i.e., 60% of identity in the binding site) as reported in the previous paragraph, the results described so far prompted us to investigate more thoroughly the inhibition mechanism of GC7 in hDHS and aDHS.

### GC7 Has No Effect on *Sulfolobus solfataricus* Cell Growth

**Figure 3:**
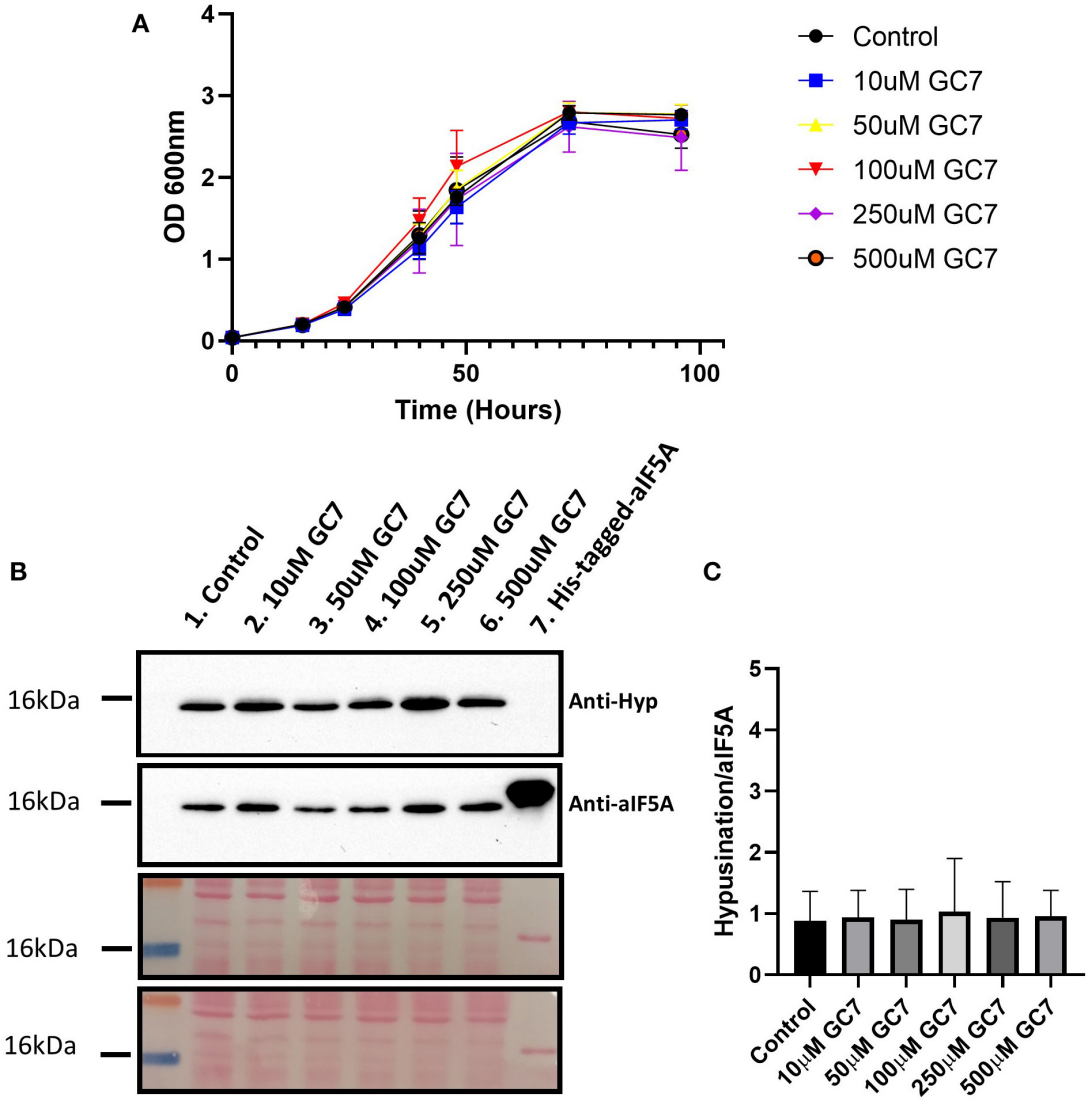
**(A)** *S. solfataricus* growth in the presence of different GC7 concentrations. *S. solfataricus* cells were grown in the absence and in the presence of 10, 50, 100, 250, and 500 μM of GC7. Growth was monitored by OD600 measurements at the indicated time points. **(B)** Western blot analysis for the detection of hypusinated aIF5A in *S. solfataricus* cells after 48 h of GC7 treatment. Lane 1, control without GC7; lanes 2–6, cells grown with the indicated GC7 concentration; Lane 7, recombinant His-tagged-aIF5A. The two membranes stained with Ponceau are reported. **(C)** Graph reporting the ratio between hypusinated over total aIF5A signals.

The MetaD results highlighted a different stability of the interaction between GC7 and DHS in *H. sapiens* and *S. solfataricus*. To experimentally confirm these theoretical results, we have tested the effect of GC7 on *S. solfataricus* cellular growth. *S. solfataricus* cells were grown in the absence and in the presence of different concentrations of GC7 (0, 10, 50, 100, 250, and 500 μM). Results presented in Figure 3A show that GC7 has no effect on the growth of *S. solfataricus* even at the highest concentration (500 μM) of the inhibitor. These results are in contrast with those obtained on eukaryotic cells (Lee et al., 2002; Schultz et al., 2018; Martella et al., 2020) and confirm the different effect of GC7 on archaeal and eukaryotic DHS enzymes, as predicted by MetaD simulations.

### GC7 Has No Effect on aIF5A Hypusination in *Sulfolobus solfataricus*

To understand if GC7 is able to inhibit the first step of the hypusination reaction in *S. solfataricus*, we have analyzed the level of hypusinated aIF5A after 48 h of treatment with different concentrations of GC7 (0, 10, 50, 100, 250, and 500μM) by Western blot. Two primary antibodies were used: one able to recognize both modified and unmodified aIF5A (Bassani et al., 2018), and a second one specifically directed against the hypusine residue.

As shown in Figure 3B, the lysates from all samples contain the hypusinated version of aIF5A as demonstrated by the presence of a signal of comparable intensity in all lanes from both anti-hypusine and anti-aIF5A, confirming that GC7 is not able to inhibit the hypusination pathway in *S. solfataricus*. The hypusination levels were represented using the ratio between the hypusination signal over that of total aIF5A (Figure 3C). These results show that GC7 has no effect on the hypusination level, confirming data obtained by the computational approach.

### Presence of GC7 Inside the Archaeal Cells

The external composition of the Archaea is characterized, in addition to the cytoplasmic membrane, by the presence of a cell wall (Albers and Meyer, 2011), which represents a structural and protective barrier that might limit uptake of external molecule like GC7, making it not available for metabolic pathways. In order to prove that GC7 can penetrate through *S. solfataricus* cell wall and therefore be present in the cytoplasm, ~40 mg of wet *S. solfataricus* pellets, treated and non-treated with 500 mM GC7, were subjected to HPLC polyamines analysis using the dansylation protocol (see materials and methods). Chromatograms showed the profile of native polyamines, visible following fluorescent derivatization, relative to Proline, Spermidine, and Spermine. Analysis of GC7 treated cells showed two additional peaks identical in retention time to those obtained following standard injection, suggesting that, despite the physical barrier represented by the cell wall, GC7 is efficiently incorporated into the cells (Supplementary Figure 5) thus confirming its inability to inhibit growth and hypusination in *S. solfataricus*.

### Molecular Dynamic Simulations Highlights the Key Differences in the Binding Site Between hDHS and aDHS

**Figure 4:**
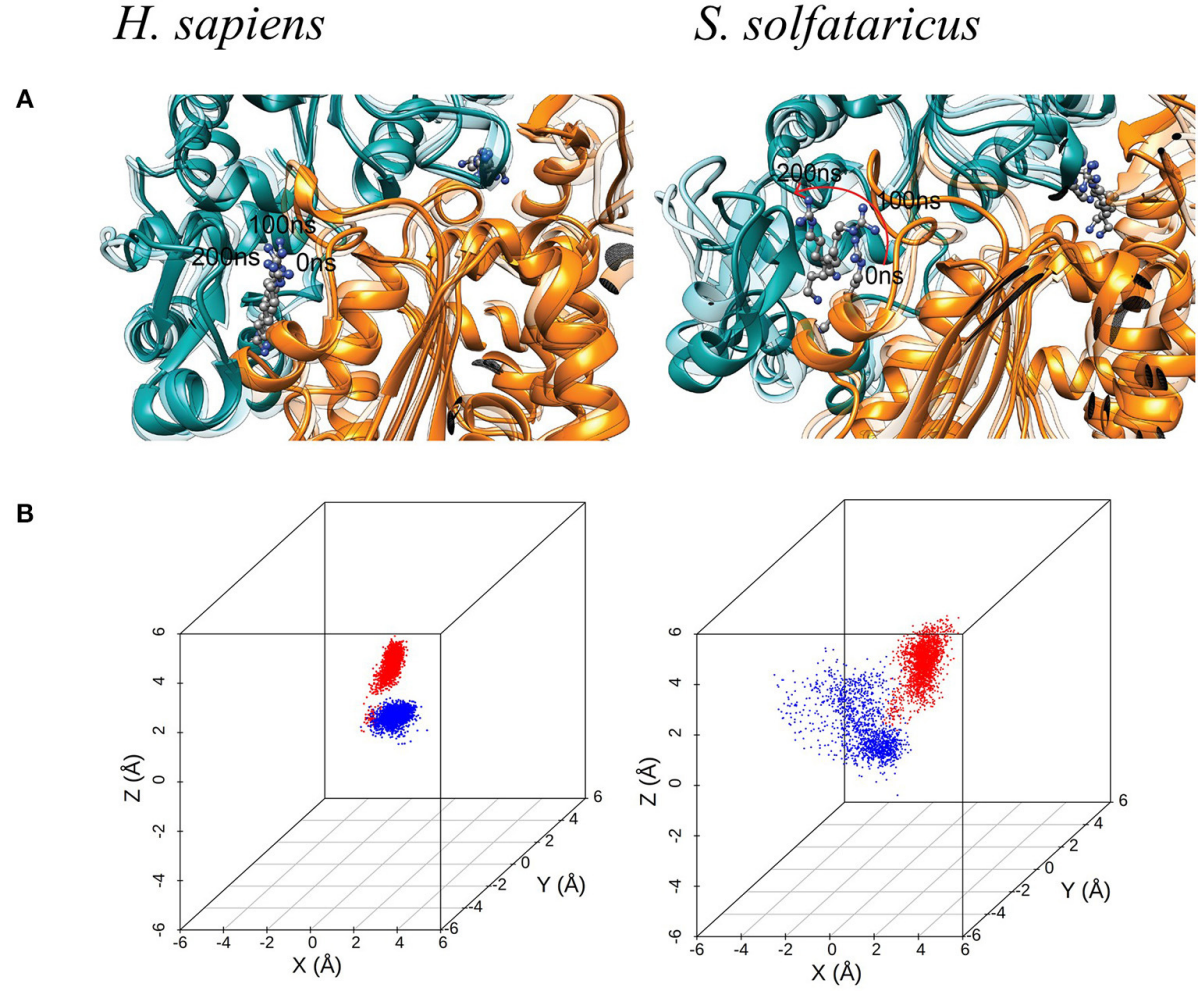
**(A)** Molecular dynamic (MD) simulation of GC7 into the DHS binding site. The MD simulation of *H. sapiens* DHS, showing the strong binding of GC7 inside the binding pocket. GC7 in *S. solfataricus* DHS presents a different interaction behavior, as shown by the drift of the molecule due to its weak interactions with the enzyme residues. The red arrow follows the movement of the molecule during the first portion of the simulation. **(B)** 3D scatterplots of GC7 center of mass coordinates shift. *H. sapiens* and *S. solfataricus* simulations are compared. The two molecules of GC7 present in each system were analyzed and represented in red (first subunit) and blue (second subunit) in the plots.

MetaD and experimental results clearly show that the GC7 is not able to inhibit the hypusination process in *S. solfataricus*. The absence of inhibitory activity of GC7 is most likely due to the different chemico-physical characteristics in the binding pockets between hDHS and aDHS. To further investigate the role of different residues in the two species, we performed unbiased MD simulations of both systems in the bound states (i.e., hDHS + GC7 and aDHS + GC7). The dimeric forms of DHS were simulated containing two binding pockets each bound to a GC7 molecule. We will refer to each binding pocket as binding site 1 and binding site 2. hDHS was simulated at 300 K (27°C), while aDHS was simulated at 348 K (75°C) to better reproduce the biological condition in which the two enzymes are active. The simulations show that GC7 is strongly bound to the human enzyme maintaining the original binding mode for the entire simulation time (Figure 4A, left side). On the contrary, in *S. solfataricus*, the GC7 does not maintain the contact with the residues in the active site during the molecular dynamic simulation, as shown in Figure 4A (right side) by the evident drift of the molecule within the binding pocket, immediately in the early stage of the simulation.

Such result is confirmed by the 3D scatterplots of GC7 COM coordinates that show the evident drift of the molecule during the *S. solfataricus* simulation (Figure 4B). Results are consistent for both GC7 molecules present in both binding sites of DHS.

Finally, to get insights on the reduced stability of GC7 within *S. solfataricus*, we compared the fluctuation of residues forming a contact with GC7 (measured as the RMSF of Cα atom of the residue). This analysis highlighted a higher fluctuation of the *S. solfataricus* binding site due to the numerous changes in the position of the ligand.

A consistent perturbation appears in the region of residues Gly196, Ser197, and Gly270, Ser271 of *S. solfataricus*, lying respectively at the two ends of GC7. This is in line with the movement of the ligand that rotate within the binding site, losing contacts at the two ends (Figure 4A).

**Figure 5:**
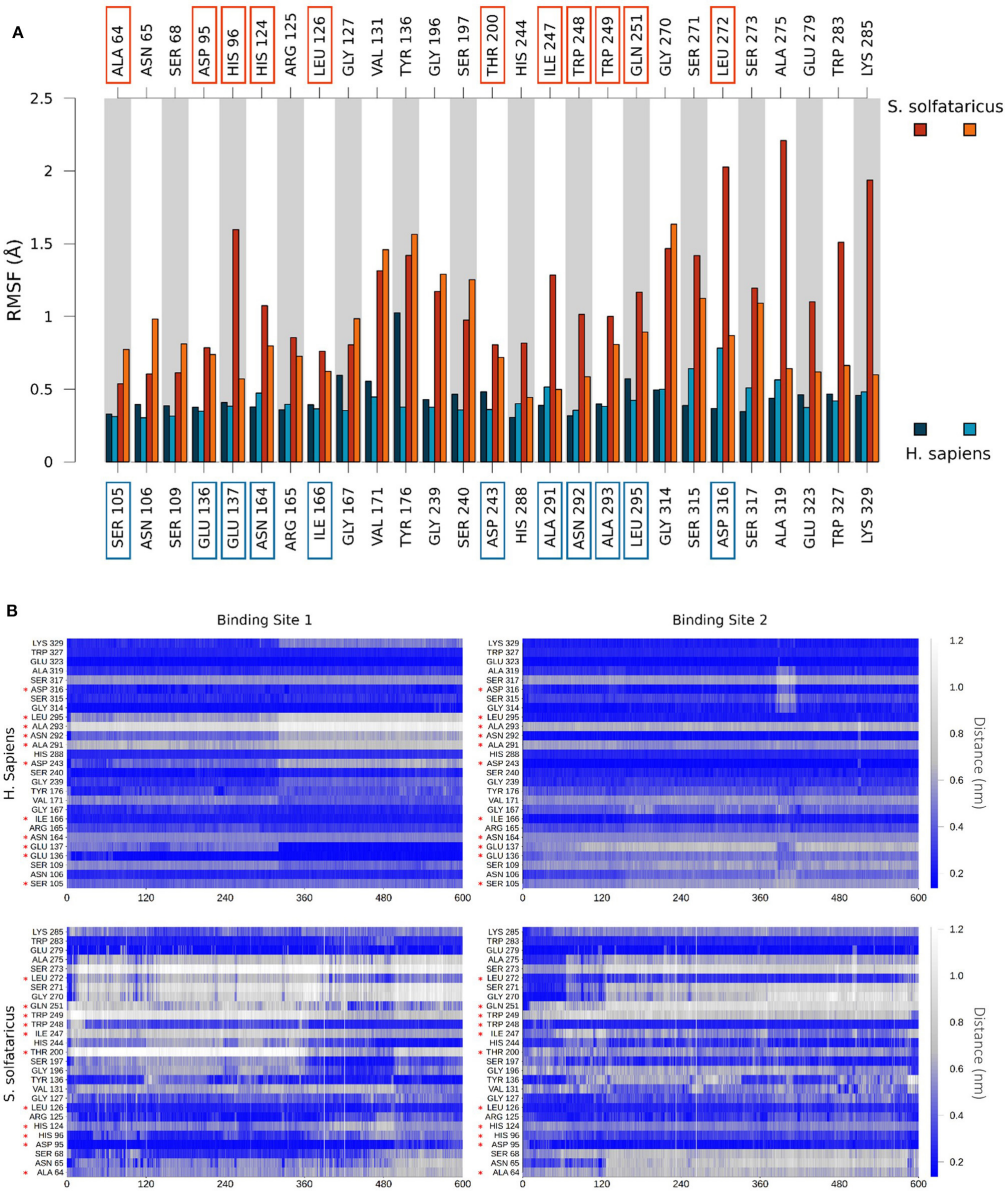
**(A)** Comparison of per-residue root mean square fluctuation of binding site residues in *H. sapiens* and *S. solfataricus*. Values are drawn as barplots in different colors for the two binding sites in *H. sapiens* (shades of blue, labeled at bottom) and *S. solfataricus* (shades of red, labeled on top). Residues not conserved in the two species are highlighted with squares. **(B)** Protein–ligand contacts during the unbiased MD simulation. The minimum distance between atoms of the ligand and atoms of each residue of the binding site was monitored for *H. sapiens* (top) and *S. solfataricus* (bottom). The values are drawn in a colorscale with blue indicating lower distances and white higher distances. Residues not conserved in the two species are highlighted with a red star.

To evaluate the stability of the protein–ligand interaction, we monitored the presence of GC7 contacts with protein residues (Figure 5B). GC7 in hDHS maintains almost all its native contacts, while in *S. solfataricus*, GC7 loses most of them during the molecular dynamic simulation, and this is a strong indication that the DHS-GC7 interaction in *S. solfataricus* is weak and unstable.

**Figure 6:**
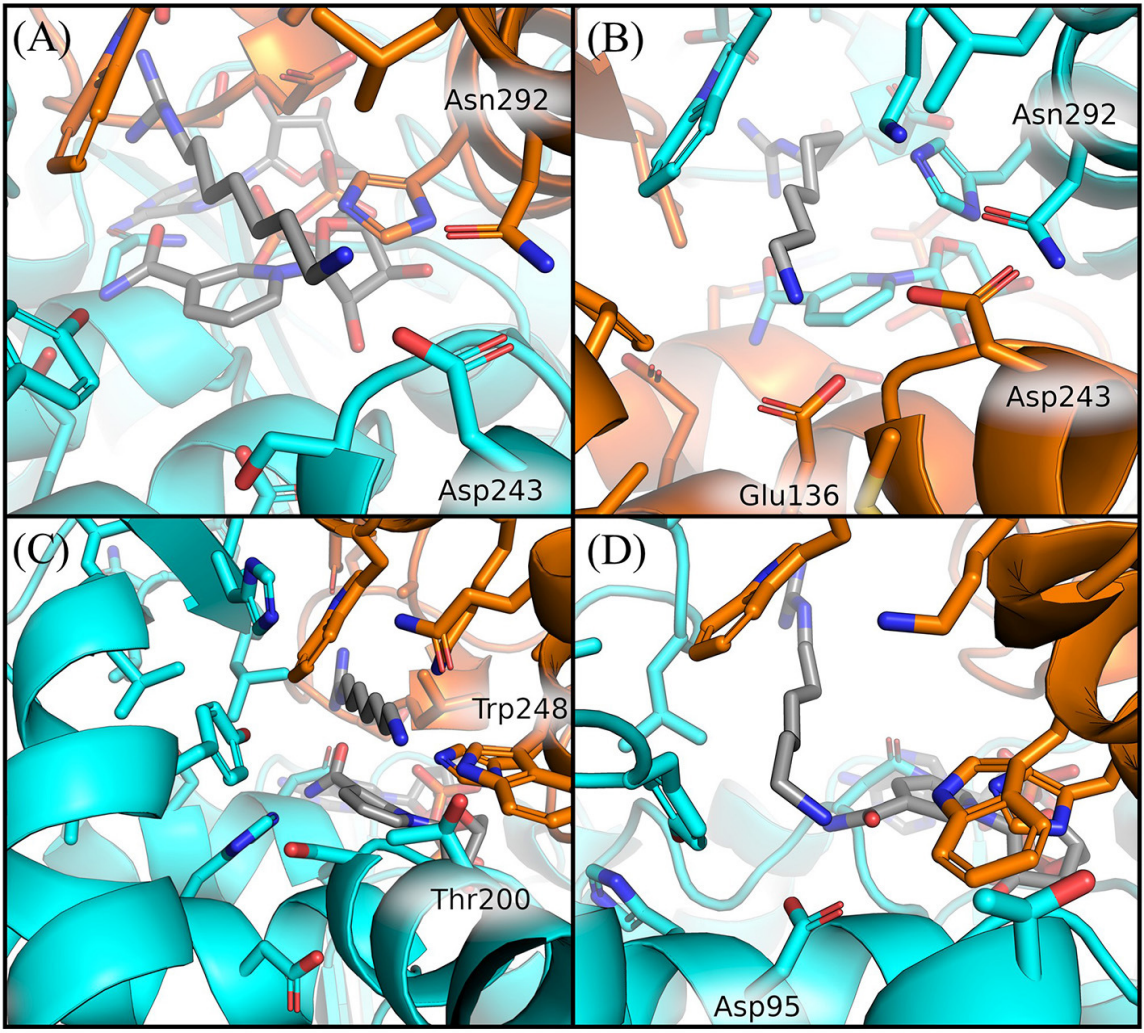
Comparison of GC7 interactions with residues of *H. sapiens* and *S. solfataricus*. Residues of *H. sapiens* within 5 Å from ligand are shown in sticks for binding site 2 at 200 ns **(A)** and for binding site 1 at 350 ns **(B)**. Residues of *S. solfataricus* within 5 Å from ligand are shown in sticks for binding site 1 at the beginning of the simulation **(C)** after 200 ns **(D)**. Protein sticks and cartoon are colored in cyan for the first chain and orange for the second chain.

In particular, we observed that GC7 in *S. solfataricus* seems more destabilized in the region of its charged terminal amine group. In *H. sapiens*, this group is involved in a salt bridge with Asp243 (Figure 5A). This interaction is highly stable in the binding site 2 and contributes to the lower ligand RMSD value registered for this ligand (Supplementary Figure 6). In binding site 1, on the other side, this interaction becomes less strong after about 310 ns, due to a shift in the terminal amine group of GC7 that start interacting with Glu136 (Figure 6B). Interestingly, the residues Asp243 and Glu136 are mutated respectively in Thr200 and Asp95 in *S. solfataricus* (Figure 6C). Therefore, in this simulation, the charged amine group of GC7 immediately moves toward Asp95 in a similar manner to the one observed for the first binding site of *H. sapiens* (Figure 6D). Overall, due to the shorter sidechain of Asp95 compared to the Glu136 of *H. sapiens*, the observed shift of the ligand is more drastic and destabilize the whole ligand binding mode. Moreover, Trp248 in *S. solfataricus* presents a very bulky sidechain that creates a steric hindrance not allowing a favorable positioning of the ligand (Figure 6C). This residue position is indeed occupied by Asn292 in *H. sapiens* that forms a dense network of h-bonds with GC7 and Asp243, further stabilizing the complex (Figure 6A).

## Discussion

Driven by the idea that knowing the molecular determinants of the GC7 binding in homologs proteins can open new frontiers for the design of inhibitors specific for either or both the hypusination process and the DHS–eIF5A complex formation, we applied a multidisciplinary and comparative approach to analyze the interaction between both human and thermophilic DHS enzymes and their inhibitor GC7. To reach our aim, we used advanced theoretical approaches, such as classical molecular dynamics or enhanced sampling [sampling (i.e., MetaD and others), successfully and widely used in the study of protein–protein interaction and mechanisms of inhibition exerted by small molecules or peptides (Di Marino et al., 2015a, Di Marino et al., 2015b; D'Annessa et al., 2019)].

The first hypusination step involving DHS is strongly conserved among *H. sapiens* and *S. solfataricus* as well as the aminoacidic sequence of the two enzymes (Bassani et al., 2018). First, to structurally compare the two proteins, we have obtained the 3D structure of *S. solfataricus* DHS through homology modeling, using the human DHS structure as template (Umland et al., 2004). Similar to the human enzyme, the archaeal protein has the active site contained within a deep narrow tunnel present at a dimer interface (Figure 2B). Interestingly, despite the high functional conservation between the two enzymes, the aDHS shows some different residues into the binding pocket. Two of them would seem to be relevant for the correct interaction between protein and ligand: (i) Asp316 in hDHS that is substituted in Leu272 in aDHS destabilizing the interaction of the GC7 guanidinium moiety; (ii) Asp243 that is presents in hDHS and it is substituted by Thr200 in aDHS destabilizing the interaction of the amine portion of the ligand. Lack of Asp243 in aDHS could be relevant for the proper interaction with GC7 since Thr200 is not able to form a salt bridge with the inhibitor.

MetaD simulations allowed us to evaluate the binding stability of the GC7 in the two different models. The data highlighted a significantly higher residence time in the hDHS compared to the *S. solfataricus* one. This result strongly underlines a lower stability of the ligand inside the thermophilic DHS binding pocket that could be explained by a different interaction network. MetaD simulations provided two different unbinding paths of the ligand. The first path (path A) occurs more frequently (i.e., 70%), and it implies a protein conformational rearrangement to allow the ligand exit (i.e., the opening of loops 310–320 and 165–170) (Supplementary Figure 4). On the other hand, the second path (path B), which was observed with a frequency of 30%, is consistent with the one previously discussed (Umland et al., 2004). In this pathway, the ligand exit occurs through the preformed tunnel that is already visible in the X-ray structures (Umland et al., 2004; Wator et al., 2020). Our MetaD calculations indicate the preference of path A, despite the conformational rearrangement, but it cannot be excluded that in unbiased simulations, binding of a ligand may occur preferentially through path B. The discovery of an alternative unbinding path may open a new possibility in the rational drug design of DHS inhibitors. The experimental approach shows that GC7 appears to be unable to inhibit *S. solfataricus* cell growth (Figure 3A) and aIF5A hypusination (Figure 3B) in contrast to what was previously described for the human enzyme (Nakanishi and Cleveland, 2016; Schultz et al., 2018; Martella et al., 2020). Considering also the instability of GC7 in the aDHS model during the MD simulations, the difference in GC7 effectiveness between human and archaeal models could depend on the different affinity for the two systems that is directly link to the diverse aminoacidic composition of the binding site. These data on an archaeal model confirm those on cellular growth (Jansson et al., 2000).

The MD simulations of the bound state of *S. solfataricus* show a higher mobility of the ligands in both binding sites, a further evidence of a formation of an unstable complex in this cell (Figure 4). The origin of the lower stability of GC7 in *S. solfataricus* can be attributed mainly to three residues different in the two species: Glu 136, Asp243, and Asn292 (Asp95, Thr200, and Trp248 in *S. solfataricus*) (Figure 6). Interestingly, all these residues are located near the terminal amine group of GC7. Asp243 plays a key role in stabilizing the GC7 inside the active site of the hDHS but not in the binding with spermidine (Wator et al., 2020). Remarkably, the first step of the hypusination reaction in *S. solfataricus* is conserved, as well as the use of spermidine as substrate of the aDHS (Bassani et al., 2018). In *S. solfataricus*, Asp243 is substituted by Thr200. This difference could explain why GC7 is ineffective for aDHS, while spermidine is correctly used as substrate by the enzyme for the first step of the hypusination reaction (Bassani et al., 2018).

Therefore, ineffectiveness of GC7 on the archaeal model may be due not only to the different growth conditions but also to the aminoacidic composition of the binding site compared to the *H. sapiens* enzyme.

The identification of the unbinding path of GC7, together with the comparative description of the structural and dynamical differences between aDHS and hDHS, provides a broad set of crucial information for the design of a new class of inhibitors, not only able to block the hypusination process but also the formation of the DHS–eIF5A complex. In the first instance, these information can be used to filter out the most promising potential inhibitory compounds during a virtual screening routine and, second, to drive the design of peptides/peptidomimetics, molecules highly suitable to inhibit protein–protein interactions (D'Annessa et al., 2020).

## Conclusion

Using a multidisciplinary approach, we have in depth-analyzed the interaction mechanism between the DHS enzyme in both human and archaeal (i.e., hDHS and aDHS). We have found that, different from hDHS, GC7 does not inhibit the activity of aDHS.

Comparative analysis has shown that aDHS lacks key residues for the GC7 interaction into the active site (Glu 136, Asp243, and Asn292 that are substituted by Asp95, Thr200, and Trp248 in *S. solfataricus*). Furthermore, we have confirmed the ineffectiveness of GC7 in inhibition of both cell growth and hypusination by experimental approaches. Moreover, MetaD simulations confirm the instability of the GC7 inhibitor within the aDHS binding pocket. These data provide a detailed knowledge of the DHS-GC7 interaction, laying the foundation for the design of new specific and more efficient DHS inhibitors.

## Data Availability Statement

The original contributions presented in the study are included in the article/Supplementary Materials, further inquiries can be directed to the corresponding author/s.

## Author Contributions

This research article was conceived by DD and AL with contributions from all authors, under the supervision of DD and AL. All authors contributed to the article and approved the submitted version.

## Conflict of Interest

The authors declare that the research was conducted in the absence of any commercial or financial relationships that could be construed as a potential conflict of interest.
