# Supplementary material for: Insights Into the Binding Mechanism of GC7 to Deoxyhypusine Synthase in Sulfolobus solfataricus: A Thermophilic Model for the Design of New Hypusination Inhibitors
Source: Front Chem. 2020 Dec 17;8:609942. doi: 10.3389/fchem.2020.609942 (PMC7773846; doi:10.3389/fchem.2020.609942)
Supplement: Supplementary file 1 [file Data_Sheet_1.docx]

Supplementary Material

##
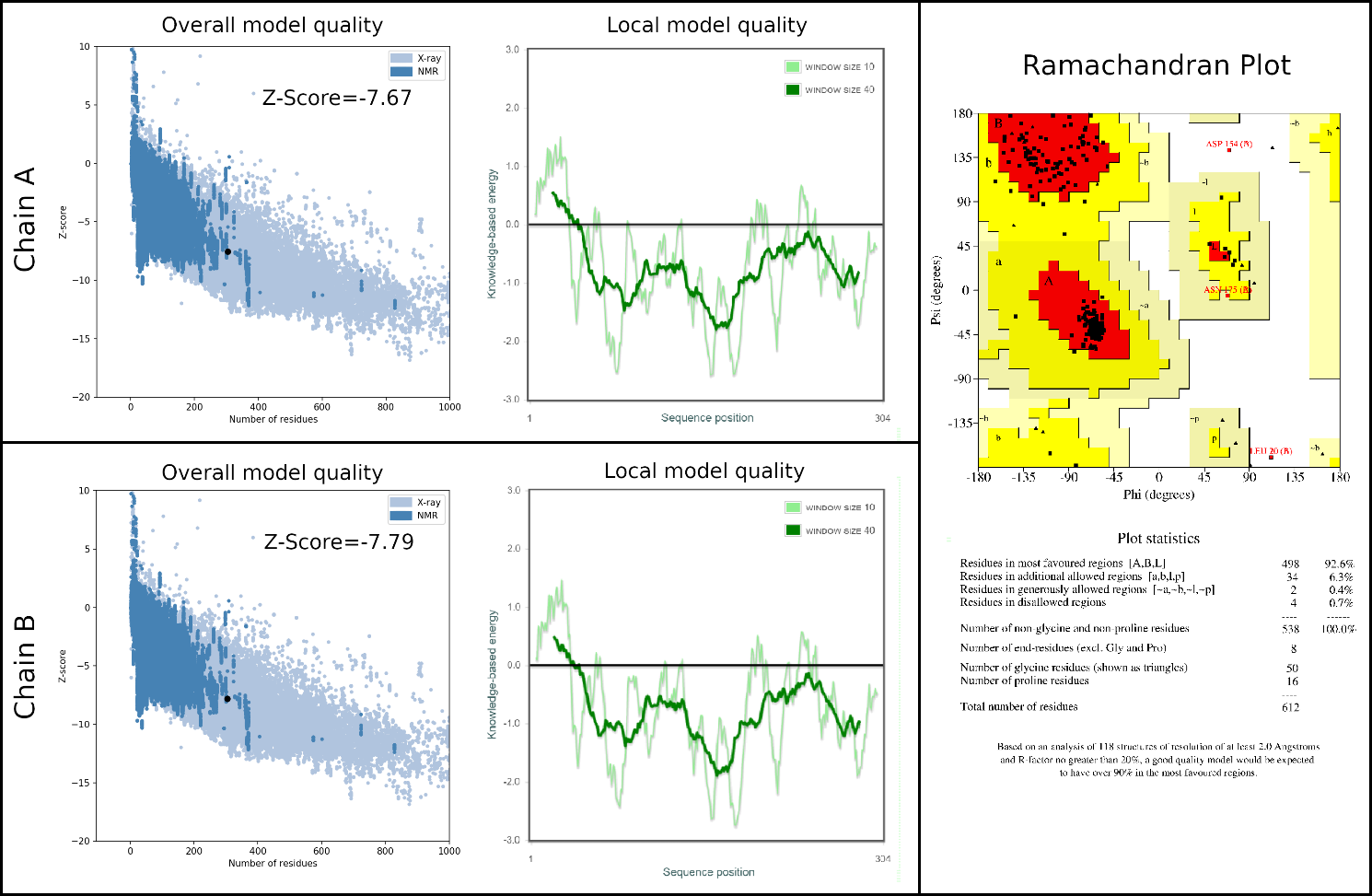
Supplementary Figures

**Supplementary Figure 1. Homology model quality assessment.** The quality of the obtained model was assessed using the ProSA validation method. The knowledge-based Z-score compared with experimental structures is shown on the left side of the figure for the two chains. In the central panel the evaluation of local model quality for the two chains is reported. The Ramachandran plot, obtained with PROCHECK, is reported on the right part of the figure.


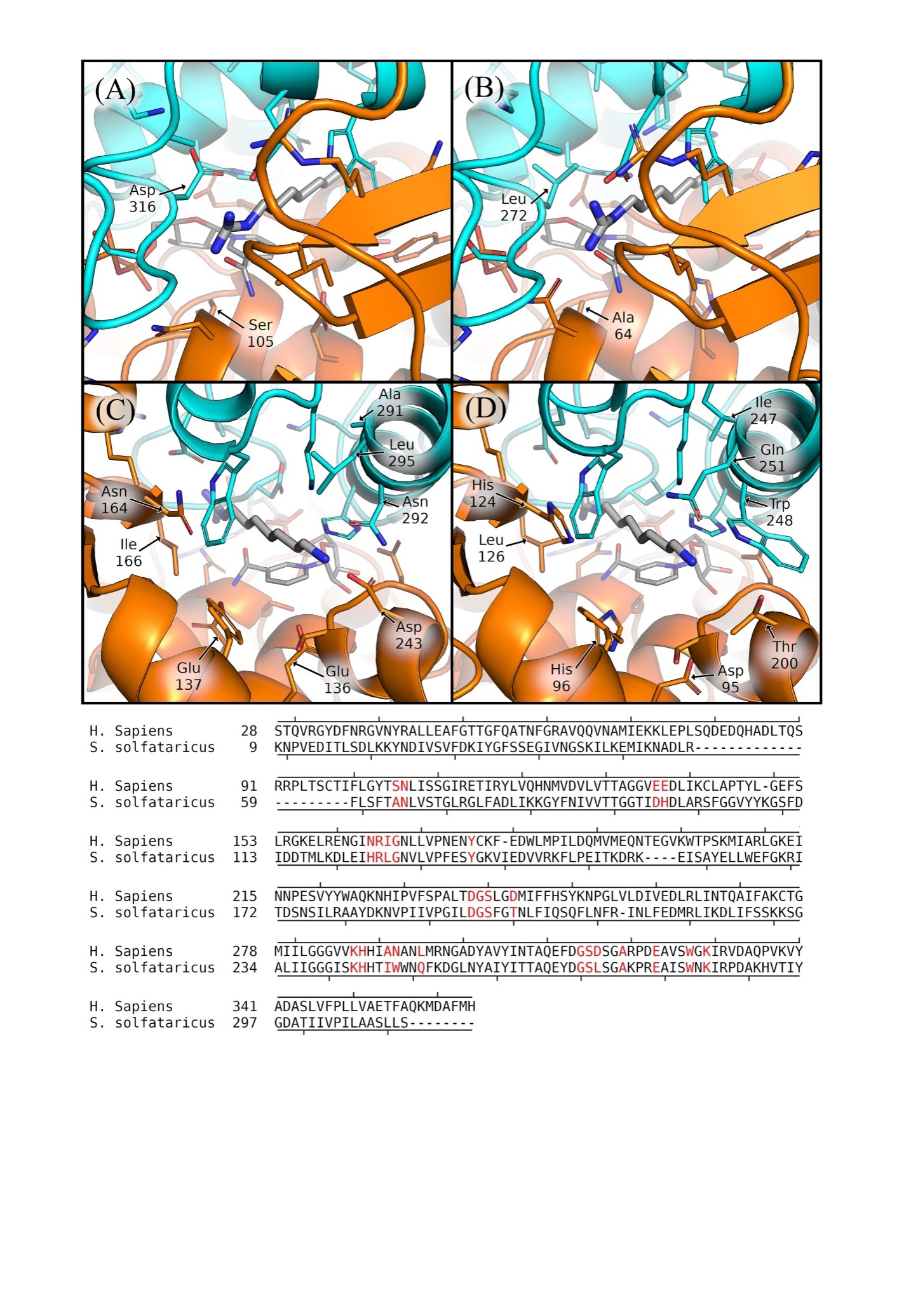


**Supplementary Figure 2. Comparison of hDHS and sDHS**. Binding site residues were compared in the hDHS structure **(A,C)** and aDHS model **(B,D)**. GC7 is shown in thick grey stick, NAD molecule in thin grey sticks. Protein residues are shown in thin sticks coloured in cyan (monomer A) and orange (monomer B). Residues that are different in hDHS and aDHS are labelled. In the bottom panel the sequence alignment between hDHS and aDHS is shown with binding site residues coloured in red.

**
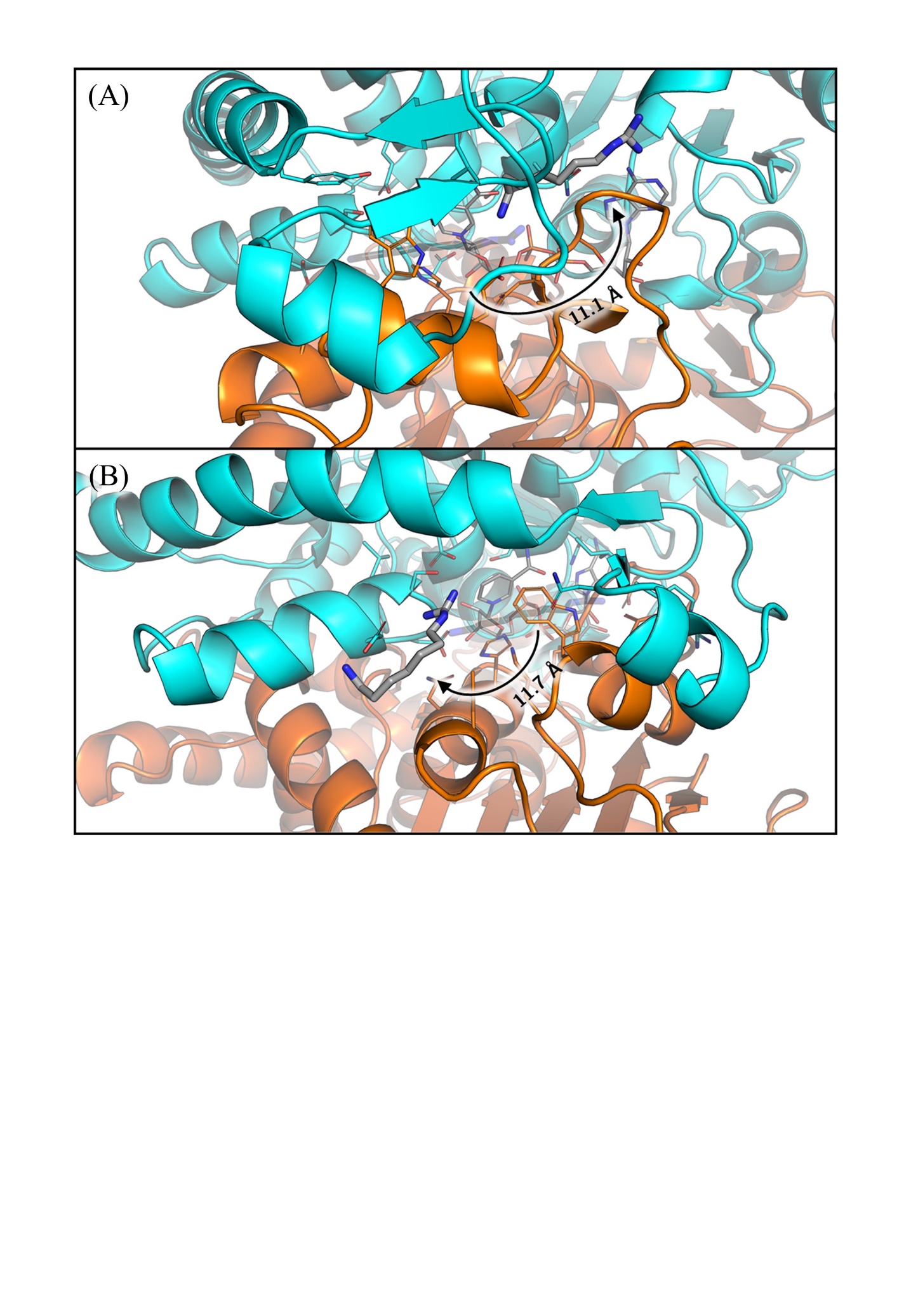
**

**Supplementary Figure 3. GC7 unbound conformations.** Two unbinding paths were found during MetaD simulations. Example of conformations where the ligand was considered unbound (number of contacts with binding site residues < 200) are reported for path A **(A)** and path B **(B)**. The ligand center of mass translation is also reported. GC7 is shown in solid thick grey stick (unbound) or transparent thick grey stick (native bound). NAD molecule is drawn in thin grey sticks. Protein residues are shown in thin sticks coloured in cyan (monomer A) and orange (monomer B).


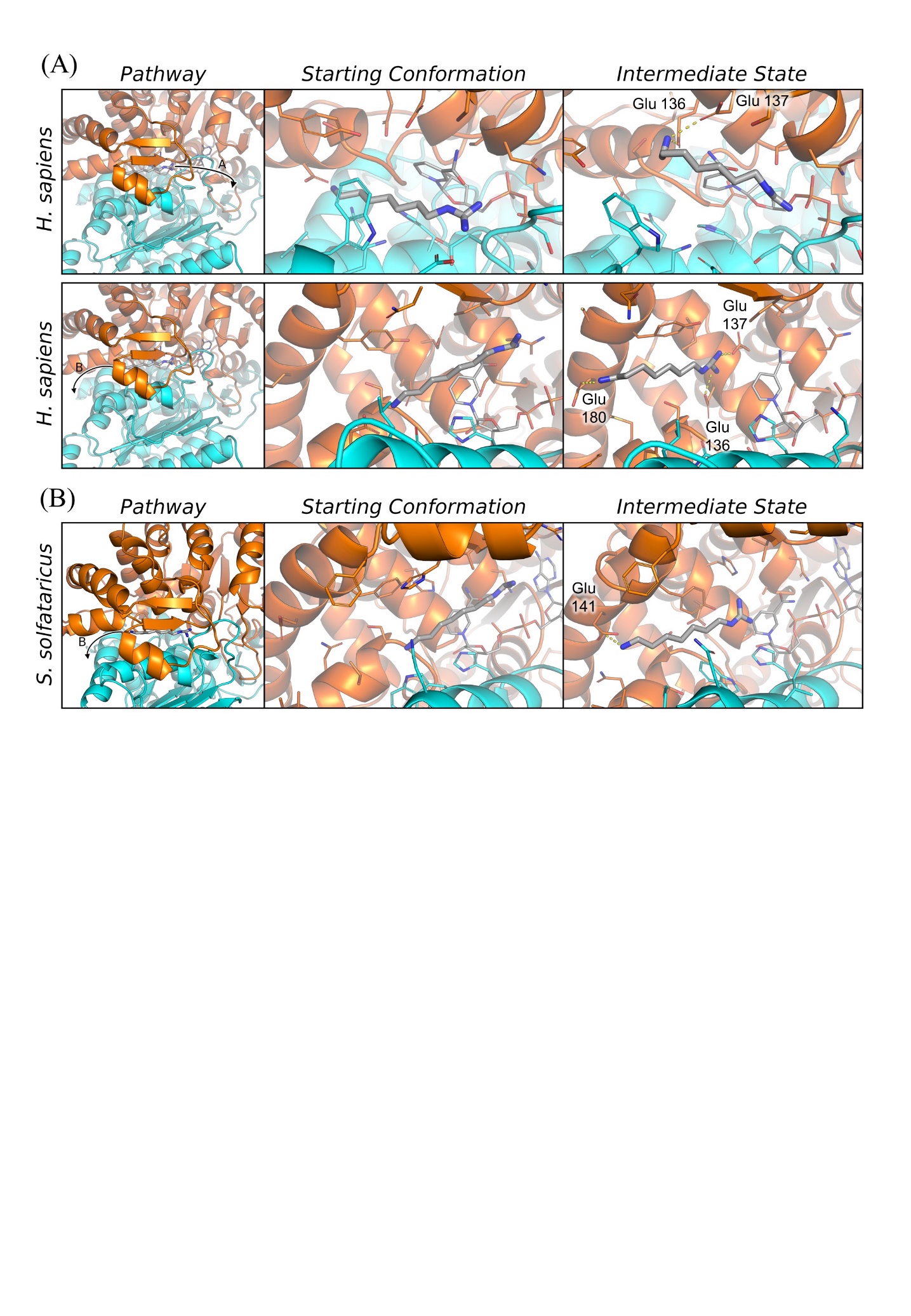
**Supplementary Figure 4. Unbinding mechanism for hDHS and aDHS in MetaD simulations. (A)** Unbinding of hDHS evolving through path A (top panel) and path B (bottom panel) with the formation of intermediate states that interact with negatively charged amino acid sidechains. **(B)** Unbinding of aDHS evolving through path B with the formation of a labile intermediate state.

**Supplementary Figure
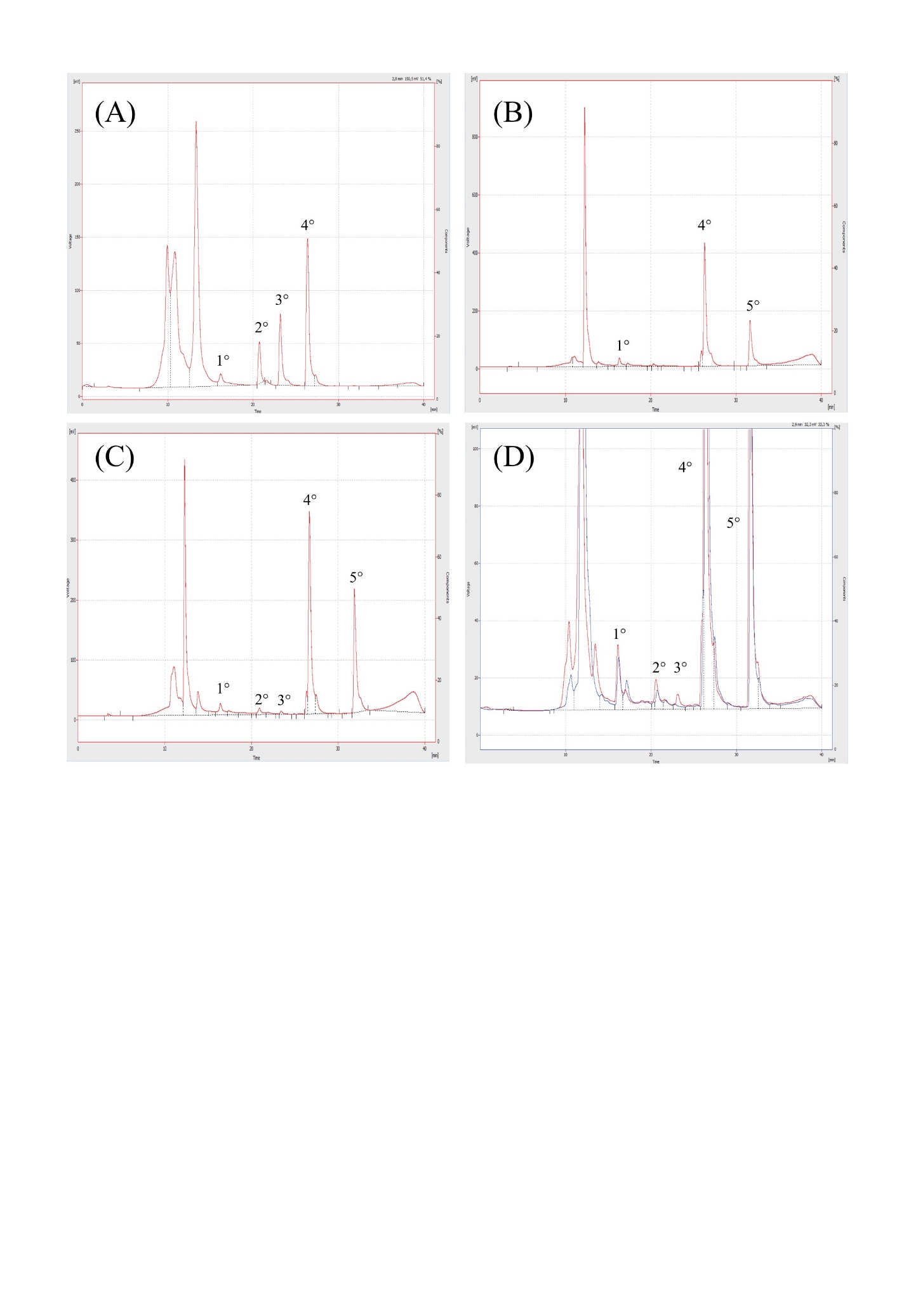
5. (A) Chromatogram of dansylated standard polyamines**. Peaks: 1= Proline; 2= GC7 1^st^peak; 3= GC7 2^nd^ peak; 4= Spermidine. For details of the separation conditions and sample preparation see material and methods. **(B) Chromatogram of dansylated polyamines of a negative control (non-treated with GC7).** Peaks: 1= Proline; 4= Spermidine; 5= Spermine. **(C) Chromatogram of dansylated polyamines of *S. solfataricus*cells treated with 500µMof GC7.** Peaks: 1= Proline; 2= GC7 1^st^ peak; 3= GC7 2^nd^ peak; 4= Spermidine; 5= Spermine. **(D) Overlay of control (non -treated with GC7) chromatogram and GC7-treated sample chromatogram, in blue and red respectively.** Peaks: 1= Proline; 2= GC7 1^st^ peak; 3= GC7 2^nd^ peak; 4= Spermidine; 5= Spermine.

**
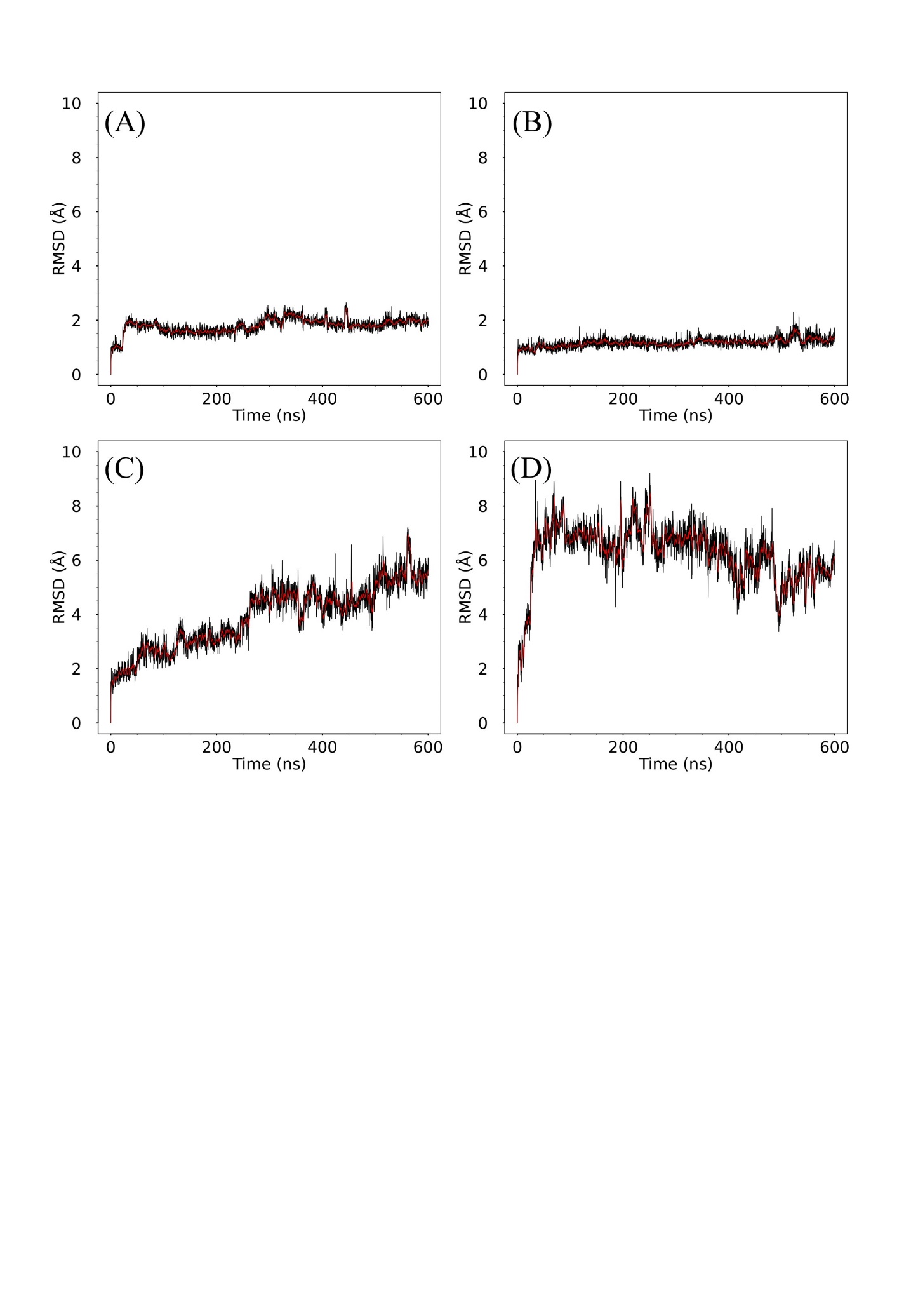
Supplementary Figure6. Ligand RMSD.** Ligand RMSD values during the unbiased MD simulations for the two binding site of *H. sapiens***(A, B)** and *S. solfataricus***(C, D)**.

**His-aIF5A**

**Hypusination**

**aIF5A**


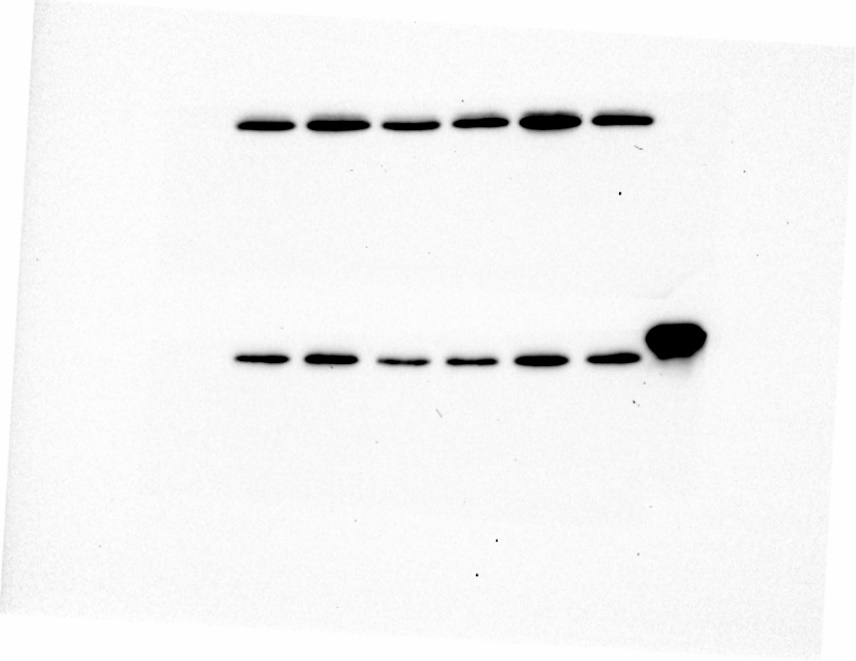


**Control**

**10µM GC7**

**50µM GC7**

**100µM GC7**

**250µM GC7**

**500µM GC7**

**Uncropped Western Blot**

**
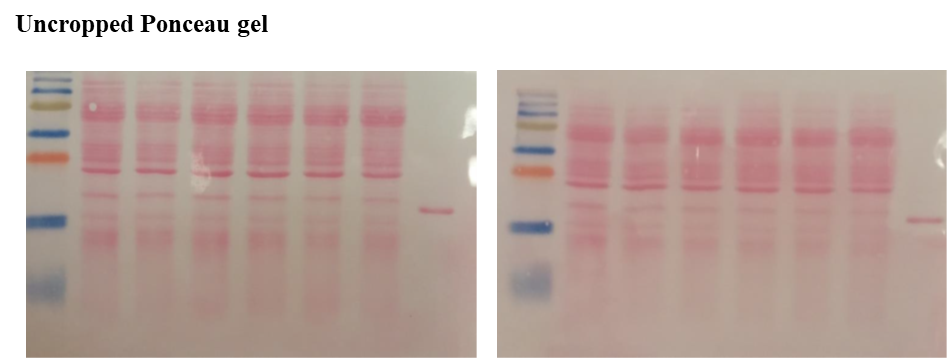
**
